# Advances in Nanoemulsion Characterization Techniques and Their Role in Oil Displacement Mechanisms

**DOI:** 10.3390/molecules31122145

**Published:** 2026-06-18

**Authors:** Ruiqi Gong, Xiaoya Feng, Min Ma, Yunlong Liu, Yuqing Li, Fanjun Shi, Xinrui Duan

**Affiliations:** 1 Xi’an Changqing Chemical Group Industry Co., Ltd. of Changqing Oilfield, China National Petroleum Corporation, Xi’an 710018, China; fxya_cq@petrochina.com.cn (X.F.); mamin_cq@petrochina.com.cn (M.M.); liyuqing1_cq@petrochina.com.cn (Y.L.); shifj_cq@petrochina.com.cn (F.S.); 2 National Engineering Laboratory for Exploration and Development of Low-Permeable Oil and Gas Fields, Xi’an 710018, China; liuyunl_cq@petrochina.com.cn; 3 Oil & Gas Technology Research Institute Changqing Oilfield Company, China National Petroleum Corporation, Xi’an 710018, China; 4 Key Laboratory of Analytical Chemistry for Life Science of Shaanxi Province, School of Chemistry and Chemical Engineering, Shaanxi Normal University, Xi’an 710119, China

**Keywords:** nanoemulsion characterization techniques, wettability alteration, interfacial tension, enhanced oil recovery, oil displacement mechanism

## Abstract

Nanoemulsions are thermodynamically unstable but kinetically stable colloidal dispersion systems with droplet sizes ranging from 20 to 500 nm. With their high specific surface area, excellent optical properties, tunable rheology, and remarkable penetration ability, these systems demonstrate enormous potential in enhanced oil recovery (EOR). This paper systematically reviews the significant advances in nanoemulsion characterization techniques and oil displacement mechanisms. The nanoemulsion characterization techniques are examined, covering a comprehensive multi-scale characterization system from particle size and distribution analysis (e.g., dynamic light scattering, laser diffraction), micro-morphology and structure visualization (e.g., transmission electron microscopy, atomic force microscopy), and interface and surface property characterization (e.g., interfacial tension measurement, zeta potential analysis) to stability and rheology assessment, as well as chemical composition and structure analysis. Furthermore, core mechanisms of nanoemulsions in oil displacement processes are briefly summarized, revealing multiple synergistic enhancement mechanisms including ultra-low interfacial tension and oil film stripping, rock wettability alteration, emulsification and viscosity reduction, improved fluid flow and injection pressure reduction. Finally, prospects for the potential application of nanoemulsion oil displacement technology in the development of low-permeability, tight, and heavy oil reservoirs are described by analyzing the current challenges such as unclear structure–activity relationships, full-chain stability (including storage, transport, injection, and reservoir aging), and environmental safety, and future research directions are pointed out, including clarifying structure–activity relationships, smart responsive system development, artificial intelligence-assisted design, and pilot-scale validation. Clarifying the link between nanoemulsion characterization techniques and oil displacement mechanisms is of significant academic and engineering value for promoting the transition from empirical application to rational design of related technologies.

## 1. Introduction

**Figure 1:**
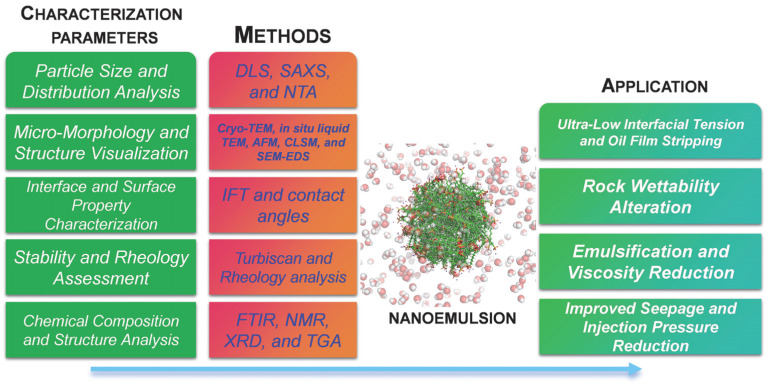
Schematic framework of nanoemulsion characterization techniques and their application in EOR.

Petroleum, often referred to as the lifeblood of modern industry, is crucial for ensuring energy security. With the gradual depletion of conventional reservoirs, the global oil and gas development focus has shifted to difficult-to-recover reserves such as low-permeability, tight, and heavy oil, for which the production proportion has exceeded 70%, but the production proportion for traditional water flooding recovery is generally lower than 30%, with a large amount of crude oil remaining as “dead oil” in rock pores in the form of thin films or clusters [1]. Chemical flooding, as an important technology for enhanced oil recovery (EOR), can improve oil recovery by reducing interfacial tension, changing wettability, etc., but has long been restricted by bottlenecks such as oversized emulsion droplet size (micron level), poor stability, weak temperature, and salt resistance, which easily block channels due to the Jamin effect in nano-scale pore throats, making it difficult to penetrate deep into reservoirs [2]. Against this backdrop, nanoemulsions have emerged as a novel nanofluid oil displacement agent. They are thermodynamically unstable but kinetically stable dispersion systems, prepared by high-energy or low-energy emulsification methods under the regulation of surfactants (and co-surfactants) with two immiscible liquid phases (usually oil and water), with dispersed phase droplet sizes precisely controlled in the range of 20–500 nm [3]. Compared with traditional microemulsions or macroemulsions, this nano-scale size endows nanoemulsions with unique physicochemical properties: long-term kinetic stability (resisting gravitational sedimentation and flocculation), optical transparency or translucency, and low viscosity [4]. More importantly for EOR applications, these properties translate into ultra-low interfacial tension, excellent wettability control, and effective emulsification and viscosity reduction [5]. The significantly increased specific surface area exponentially improves the spreading efficiency of active components on rock surfaces, while their nano-scale droplets enable them to penetrate micro–nano-scale pore throats that conventional chemical agents cannot reach, making them a rapidly advancing research frontier [4,5]. With increasingly depleted global conventional oil and gas resources and growing energy supply–demand contradictions, the efficient utilization of unconventional oil and gas resources (including low-permeability, tight, and heavy oil reservoirs) has become a strategic focus for ensuring national energy security [6]. However, these reservoirs generally have inherent problems such as small pore throat sizes, extremely low permeability, strong heterogeneity, high crude oil viscosity, or strong oil–water–rock interface interactions, leading to low traditional water flooding efficiency, with a large amount of crude oil remaining in the deep reservoir [7]. As an emerging nano-intelligent oil displacement strategy, nanoemulsion technology provides a revolutionary approach to break through the above technical bottlenecks by delivering active components such as surfactants, solvents, and functional nanoparticles to the deep reservoir in the form of “nano-inclusions” and achieving precise, efficient synergistic effects at the oil–water–rock three-phase interface [8]. Specifically, nanoemulsions reduce oil–water interfacial tension, promote wettability alteration toward more water-wet conditions, and improve seepage capacity through nano-scale effects. Together, these mechanisms enable the effective recovery of residual oil in micro–nano-scale pores [9]. In recent years, with the deep integration of nanotechnology, interface chemistry, and multi-scale simulation methods, research on nanoemulsions in the field of enhanced oil recovery (EOR) has shown explosive growth [10]. Since 2018, the number of global related research papers has increased exponentially, with research categories evolving from early formula optimization and macroscopic performance evaluation to detailed characterization of nanoemulsion bulk properties, dynamic behavior simulation under reservoir conditions, and development of smart responsive nanoemulsions [11]. Especially in terms of characterization techniques, the application of multi-scale characterization methods such as dynamic light scattering (DLS), small-angle X-ray scattering (SAXS), and cryo-transmission electron microscopy (Cryo-TEM) has enabled precise analysis of nanoemulsion particle size distribution, interface structure, and stability mechanisms at the molecular-nano scale [12,13,14,15]. Meanwhile, oil displacement mechanism studies based on in situ observation and numerical simulation have revealed multiple synergistic mechanisms such as structural disjoining pressure, emulsification and viscosity reduction, and intelligent release, providing a theoretical foundation for reservoir-adaptive design of nanoemulsions [16,17,18]. The schematic framework of this review is represented in Figure 1. However, the current research still faces severe challenges such as insufficient long-term stability under high-temperature and high-salinity environments, unclear interaction mechanisms between nanoemulsions and complex reservoir fluids, and high costs for large-scale preparation, urgently requiring deep integration of characterization techniques and oil displacement mechanism research to promote technological breakthroughs. Although multi-scale characterization and displacement mechanisms are fundamental to nanoemulsion EOR, the extent to which current characterization results actually validate the proposed mechanisms remains insufficiently explored. To address this, this review systematically evaluates the correspondence between characterization techniques and mechanisms. Clarifying this alignment is essential to transition the field from observations to validated mechanistic understanding, thereby exposing methodological bottlenecks and directing future research.

## 2. Advances in Nanoemulsion Characterization Techniques: From Macroscopic Properties to Microscopic Structure

Accurate and comprehensive characterization is the foundation for understanding, designing, and optimizing nanoemulsion systems. In recent years, characterization techniques have developed from single particle size analysis to multi-parameter, multi-scale comprehensive analysis, providing more-comprehensive technical support for the research, development, and application of nanoemulsions.

### 2.1. Particle Size and Distribution Analysis

Droplet size and its distribution are core parameters determining nanoemulsion stability, optical properties, and transport performance, and accurate characterization is the prerequisite for optimizing system design. In recent years, research in this field has evolved from the independent application of single techniques to multi-method combination and collaborative verification, significantly improving the reliability of characterization results. Dynamic light scattering (DLS) is the standard technique for measuring the hydrodynamic diameter of nanoemulsions, analyzing scattering light intensity fluctuations caused by Brownian motion. It offers advantages of rapidity and non-destructiveness, while simultaneously providing the polydispersity index (PDI) to evaluate system uniformity. To improve the accuracy of DLS, a multiple-angle dynamic light scattering (MDLS) apparatus was developed to enhance measurement accuracy and to eliminate long-range interactions and intraparticle interference via extrapolation, extending the application range from nanoparticles to submicron particles [19]. To address the integral equation challenge in dynamic light scattering (DLS) for nanoparticle sizing, a regularization inversion algorithm was developed, which demonstrated accurate nanoparticle size distribution measurement, thereby advancing DLS-based particle sizing technology [20].

On this basis, DLS were used to systematically study the influence of gemini surfactant–polymer–nanoparticle (SPN) ternary composite systems on oil droplet size: oil droplet size in pure surfactant systems (14-6-14 GS) was 6.8–32 nm, increasing after adding partially hydrolyzed polyacrylamide (PHPA), while further introducing SiO_2_ nanoparticles caused oil droplet size to fall back to the nano-scale range, revealing the steric hindrance effect between components [21]. Meanwhile, Xu et al. investigated the long-term stability of nonionic surfactant-stabilized nanoemulsions under high-temperature conditions using DLS, finding that, even after 30 days at 65 °C, the system particle size remained stable at around 15 nm, indicating good anti-aging ability [22]. These studies not only validate the applicability of DLS technology but also highlight its key role in real-time monitoring of nanoemulsion structure evolution.

Furthermore, small-angle X-ray scattering (SAXS), with its unique advantage of providing statistical information on droplet size, shape, and interface layer thickness within the 1–100 nm scale, has become a powerful tool for analyzing the fine structure of interface adsorption layers. Previous studies have quantitatively analyzed the adsorption behavior of nanoparticles at emulsion interfaces using SAXS, establishing a structure–activity relationship between interface layer thickness and system stability [23,24]. In response to the accurate characterization needs of non-spherical and polydisperse systems, emerging nanoparticle tracking analysis (NTA) technology provides more-precise particle size distribution information than traditional DLS by real-time tracking the Brownian motion trajectories of individual droplets. Based on NTA, a new methodology to determine size differences of nanoparticles were developed to determine which sizes of nanoparticles are statistically significant between two groups of samples [25]. In addition, field flow fractionation (FFF) technology can effectively separate different components in complex systems based on particle size, density, and surface charge, opening up new avenues for the independent characterization of multi-component systems [26].

### 2.2. Micro-Morphology and Structure Visualization

Despite the advances in particle size analysis techniques, the intrinsic physicochemical properties of nanoemulsions impose significant limitations on conventional microscopic analysis techniques, including electron microscopy. Furthermore, there is a lack of effective methodologies to investigate the relationship between nanoemulsion properties and their performance. In the petroleum industry, research and characterization of nanoemulsions primarily rely on techniques such as DLS, TEM, and SEM. While TEM and SEM can provide nano-scale information, the vast majority of microscopy studies to date have been largely limited to particle size measurements of nanoemulsions, thus overlapping with the capabilities of DLS. Nevertheless, imaging techniques provide irreplaceable intuitive evidence for in-depth understanding of nanoemulsion microstructures by directly revealing their spatial morphology and distribution characteristics. Among them, transmission electron microscopy (TEM) and cryo-electron microscopy (Cryo-TEM) technologies have become core means of morphology characterization with their nano-scale resolution. Cryo-TEM technology effectively avoids artificial artifacts such as droplet deformation introduced during traditional TEM drying processes through rapid-freezing sample preparation, enabling a more authentic reflection of the intrinsic structure of emulsions in the liquid state. However, DLS and TEM/SEM provide fundamentally distinct yet complementary information: DLS yields ensemble-averaged hydrodynamic diameters and size distributions, whereas microscopy resolves individual droplet morphology, spatial distribution, and internal structure. Crucially, microscopy-based characterization is susceptible to various sample-preparation artifacts, including dilution-induced structural changes, drying artifacts (conventional TEM), staining-induced aggregation, ice crystal formation (cryo-fixation), substrate–droplet interactions, and electron beam damage. Because these microscopic artifacts can severely skew perceived size and morphology, DLS is required to provide a statistical baseline. Conversely, microscopy is needed to validate the physical reality behind DLS averages. Therefore, the integration of scattering and microscopy techniques is essential for reliable nanoemulsion characterization.

Cryo-TEM plays a critical role in evaluating perfluorocarbon nanoemulsion stability by overcoming DLS limitations. While DLS alone cannot detect particle size changes and may mislead stability assessment, Cryo-TEM reveals detailed particle evolution through cryo-fixed sample imaging. The combination of both techniques provides comprehensive understanding, advancing analytical tools for clinically applicable nanoemulsions [27]. A principle directly applicable to EOR nanoemulsion characterization. Cryo-TEM was used to identify the structures formed, revealing that small oblate micelles arrange into wormlike aggregates rather than forming conventional wormlike micelles [28].

Recently, in situ liquid TEM has been offering significant advantages over cryo-EM by preserving nanoparticles in their native liquid environment without drying or freezing artifacts. While cryo-EM compromises the ability to observe dynamic processes and may modify sample structure, in situ liquid TEM enables real-time imaging of dynamic processes in liquid conditions. This approach achieves atomic-scale resolution without requiring surface modification; allows comprehensive characterization through combined imaging, spectroscopy, and diffraction techniques; and maintains the true structure of both metallic and biological samples, providing more-accurate and representative results [29]. Liquid-phase TEM revealed anomalous non-Brownian diffusion in emulsion droplets, with sub- and super-diffusive motion arising from droplet–surface interactions and electron beam-induced fractal energy landscapes [30].

Atomic force microscopy (AFM) technology, with its ability to conduct three-dimensional morphology scanning and mechanical property measurement of samples in near-natural states, serves as a powerful supplement for in situ characterization. AFM enabled measurement of micro-sized water droplet contact angles on rough quartz surfaces in natural sandstone, overcoming limitations of traditional optical methods for micro-scale mineral grains. It revealed asymmetric droplets with contact angles varying along triple-phase lines (27.8–50.3°) due to surface roughness, heterogeneity, and atomic arrangement [31]. AFM analysis characterized the morphology and size distribution of nanoemulsion droplets, revealing that oil droplets were mostly spherical or semi-spherical with uniform size distribution, complementing other characterization methods to validate the successful development of stable MH-loaded nanoemulsions [32]. Although this system is a pharmaceutical nanoemulsion, it is included as a methodological analogy demonstrating AFM’s capability to resolve spherical and semi-spherical droplet morphology and validate size distributions obtained by DLS. In addition, scanning electron microscopy combined with energy dispersive spectroscopy (SEM-EDS) can simultaneously obtain surface morphology and element distribution information [33], while confocal laser scanning microscopy (CLSM) realizes dynamic tracking of emulsion migration behavior in porous media through three-dimensional fluorescence imaging [34]. These technologies together construct a multi-dimensional characterization platform for nanoemulsion structure–activity relationship research.

Microscopy techniques (TEM, Cryo-TEM, liquid TEM, AFM) provide complementary morphological and mechanical information that cannot be obtained from scattering methods alone, while each technique exhibits distinct sample preparation artifacts that must be carefully considered.

### 2.3. Interface and Surface Property Characterization

Interface and surface properties directly determine the oil displacement efficiency of nanoemulsions in reservoirs and are core indicators for evaluating their application potential. The current research has deepened from static parameter measurement to dynamic behavior analysis and synergistic effect evaluation. Significant reduction in interfacial tension (IFT) is the key for nanoemulsions to achieve efficient oil displacement. The synergistic enrichment of nanoparticles and surfactants at the oil–water interface can not only reduce IFT to below 0.1 mN/m but also maintain long-term stability by enhancing interface film strength. Zhang et al. reported that nanoemulsion systems can still maintain oil/water IFT at the 10^−2^ mN/m level under high-temperature and high-salinity conditions, demonstrating their adaptability to harsh environments [35].

The imbibition and displacement performance of chemical systems correlates directly with their interfacial tension (IFT) reduction capacity, demonstrating a clear gradient: microemulsion-forming systems achieve the lowest IFT and superior performance, followed by nanoemulsions, whereas conventional surfactants exhibit higher IFT values [36]. This IFT regulation serves as the core mechanism for effective chemical flooding; even under extreme high-temperature and high-salinity conditions, achieving ultra-low IFT can significantly enhance oil stripping and emulsification, thereby leading to substantial incremental oil recovery [37].

Zeta potential analysis serves as an important means to predict emulsion electrostatic stability, with absolute values higher than 30 mV usually indicating sufficient repulsion to maintain stability. For example, 14-6-14 GS-stabilized systems showed a decline from +42.7 mV (1 h) to +11.0 mV (30 days), reflecting weakened electrostatic repulsion and decreasing stability. In other systems, surfactant–polymer (SP) nanoemulsions maintained low values (initial +5.6 mV, 30-day +0.3 mV) due to steric hindrance, while surfactant–polymer–nanoparticle (SPN) systems exhibited negative potentials (initial −30.2 mV, 30-day −13.2 mV) from silica nanoparticle adsorption, with potential changes correlating to stability shifts [21].

Wettability alteration ability is another key factor determining oil washing efficiency. For example, the wettability alteration capability could be evaluated by measuring contact angles between synthetic oil and core slices after different fluid treatments—with the initial contact angle of approximately 23.8° (after two weeks of aging) shifting to 27.5°, 147.0°, and 156.6° respectively after 4 h of exposure to formation water, complex surfactant, and nanoemulsion, which transformed rock surfaces from hydrophobic to hydrophilic, converted capillary force from a resistance into a driving force, facilitated injected fluid penetration into pore throats, and enhanced crude oil detachment [22].

In addition, interface shear rheology evaluates interface film strength by measuring viscoelastic parameters at oil–water interfaces [38]. Interfacial rheology serves as a valuable tool for designing more-efficient surfactant formulations in EOR, as it helps optimize the rigidity and viscoelastic properties of interfaces, thereby promoting the formation of continuous phase threads that are easier to sweep and enhancing oil recovery. Additionally, it provides insights into intermolecular interactions between surfactants and asphaltenes, as well as the impact of surfactant concentration on interface viscosity/elasticity.

### 2.4. Stability and Rheology Assessment

It is important to recognize that the characterization techniques discussed above face significant limitations under reservoir-relevant conditions. DLS and zeta potential measurements typically require substantial sample dilution, which can alter the ionic strength, surfactant adsorption equilibria, and electrical double-layer structure surrounding nanoemulsion droplets, potentially yielding size and stability parameters that differ from those under undiluted reservoir conditions. As demonstrated by Pal et al. [21], the zeta potential of gemini surfactant-stabilized nanoemulsions declined from +42.7 mV to +11.0 mV over 30 days, highlighting how electrostatic stability parameters are sensitive to time-dependent adsorption equilibrium changes that may be further perturbed by dilution. Crude-oil opacity and multiple scattering further compromise optical-based sizing methods, while injection-relevant shear rates—often orders of magnitude higher than standard rheometer protocols—may not be adequately captured in laboratory viscosity and viscoelasticity assessments. Zhang et al. [35] reported that nanoemulsion systems achieved ultra-low IFT at the 10^−2^ mN/m level and maintained emulsion stability at temperatures up to 100 °C and salinities of 39,000 mg/L, yet the long-term rheological behavior under simultaneous high-pressure, high-temperature, and high-shear conditions remains difficult to reproduce in standard laboratory setups. Furthermore, Zhong et al. [39] showed that high reservoir pressure significantly affects the rheological properties of polymer solutions used in chemical flooding, suggesting that similar pressure-dependent effects on nanoemulsion rheology may be overlooked in atmospheric-pressure measurements. These methodological gaps highlight the need for in situ characterization techniques and pilot-scale validation under representative reservoir conditions, as further discussed in Section 4.

### 2.5. Chemical Composition and Structure Analysis

Chemical composition and intermolecular interaction analysis are important theoretical foundations for optimizing nanoemulsion formulas and realizing rational design. Fourier-transform infrared spectroscopy (FTIR) and nuclear magnetic resonance (NMR) technologies can effectively identify system components and reveal intermolecular interaction mechanisms by recognizing characteristic functional groups and chemical shifts. FTIR analysis is central to understanding the interfacial materials and molecular interactions in water-in-oil emulsions for EOR applications [40]. Also nuclear magnetic resonance was widely used to study the EOR mechanism from the level of pore structures. Since the NMR signal of oil is easily detectable in a magnetic field, while D_2_O does not have a significant signal, the T2 spectrum was used to observe the changes in oil saturation and the distribution of oil in pores [41].

Thermogravimetric analysis (TGA) can quantitatively evaluate the thermal stability and relative content of each component through program-controlled temperature and mass loss monitoring. For instance, TGA has been utilized to evaluate the thermal stability of shape-modified silica nanoparticles for oil-wet sandstone reservoirs, providing critical data for predicting their high-temperature survival boundaries [42]. Although X-ray diffraction (XRD) is currently less utilized in the EOR nanoemulsion literature than in other fields, it provides valuable insights for analyzing the crystal structure of solid nanoparticles in emulsion systems. As a methodological analogy from food-preservation research, chitosan/pullulan composited essential oil nanoemulsions [43] demonstrate that XRD can confirm the intermolecular interactions and structural integrity of polymer–nanoparticle composites; this cross-disciplinary analytical approach is directly transferable to validating the structural basis of solid-laden interfaces in reservoir Pickering nanoemulsions.

A comprehensive comparison of nanoemulsion characterization techniques is represented in Table 1.

## 3. Core Role of Nanoemulsions in Oil Displacement: Multi-Scale Synergistic Enhancement

The oil displacement effect of nanoemulsions is not a single mechanism but a com-plex system with multiple physicochemical processes synergistically enhancing at the pore scale. The characterization methods described above collectively provide a detailed understanding of nanoemulsion systems. The performance of nanoemulsions in oil displacement, e.g., ultra-low interfacial tension, oil film stripping, rock wettability alteration, emulsification and viscosity reduction, and improved seepage and injection pressure reduction, is intrinsically governed by their physicochemical properties. Key examples include: the reduction in water–oil–rock multiphase interfacial tension by surfactants; the partial intercalation of nanoemulsion oil phases into resins or asphaltenes, thereby reducing viscosity and pressure; and the simplification of operational procedures through the construction of smart responsive systems.

To rigorously connect characterization with EOR mechanisms, it is critical to elucidate the underlying thermodynamic and kinetic principles while clearly distinguishing between experimentally validated phenomena and mechanistic interpretations proposed in the literature.

### 3.1. Ultra-Low Interfacial Tension and Oil Film Stripping

The primary mechanism by which nanoemulsions mobilize residual oil is the drastic reduction in capillary resistance, governed by the Young–Laplace equation. The enrichment of surfactants at the oil–water interface lowers the interfacial tension (IFT) to ultra-low levels (mN/m), which has been robustly confirmed by tensiometry and microscopic visualization [45]. Beyond capillary de-trapping, low IFT fundamentally alters the thermodynamic state of the oil film. According to the Dupré equation, the work of adhesion is significantly reduced, shifting the balance from adhesion to cohesion. This thermodynamic shift drives the spontaneous thinning, rupture, and stripping of the oil film from pore walls, a process that has been directly observed in micromodel experiments, particularly within small pore throats [46].

### 3.2. Rock Wettability Alteration

In the rock wettability alteration process, the final wetting state is dictated by the synergistic coupling of rock mineralogy, brine salinity, and surfactant/nanoparticle adsorption dynamics. For instance, mineral-specific surface charges (e.g., positively charged carbonates vs. negatively charged sandstones) govern the electrostatic adsorption modes of ionic surfactants. Meanwhile, brine salinity modulates the electrical double layer thickness, compressing it at high salinities to either promote or hinder surfactant/nanoparticle approach and adsorption [47]. Furthermore, at the micro-/nano scale, reservoir rock surfaces are inherently rough. This roughness leads to contact angle hysteresis, which quantifies the difference between advancing and receding angles, meaning the effective wettability cannot be defined by a single static contact angle. While AFM visualization has experimentally confirmed the micro-morphology of adsorbed surfactant/nanoparticle layers on rock surfaces [48], the precise molecular configurations dictating the transition between Wenzel and Cassie–Baxter wetting states remain largely theoretical interpretations that require further mechanistic validation.

### 3.3. Emulsification and Viscosity Reduction

The viscosity reduction mechanism operates across macroscopic emulsification and microscopic solvent–oil interactions. At the macroscopic level, nanoemulsions act as efficient emulsifiers, dispersing continuous high-viscosity crude oil into discrete O/W droplets, thereby improving mobility. The Jamin effect of these droplets also selectively blocks high-permeability channels, enhancing macroscopic sweep efficiency. At the microscopic level, for heavy oil, the mechanism is intrinsically linked to crude oil composition. The high viscosity of heavy oil originates from the colloidal network formed by asphaltenes and resins, stabilized by π-π stacking and hydrogen bonding. Organic solvents in nanoemulsions (e.g., limonene) possess matching solubility parameters, allowing them to penetrate the heavy oil matrix and interact specifically with these asphaltene aggregates. The current physicochemical evidence, primarily from rheology and spectroscopy, supports the proposed interpretation that these solvents interfere with π-π stacking and disrupt the hydrogen-bonding networks, collapsing the colloidal structure and reducing bulk viscosity. Concurrently, nanoparticles stabilize the newly formed interfaces via Pickering effects, preventing droplet coalescence and enhancing viscosity reduction efficiency [49].

### 3.4. Improved Seepage and Injection Pressure Reduction

In low-permeability and tight reservoirs, the nano-scale size of droplets ensures they can penetrate deep into micro/nano-pores without causing mechanical plugging. The reduction in injection pressure is both a fluid-dynamic and an interfacial phenomenon: the elimination of capillary resistance and the stripping of boundary layer fluids reduce the starting pressure gradient. This seepage improvement has been experimentally verified using nuclear magnetic resonance (NMR), which quantified the mobilization of crude oil from small pores and the resulting increase in effective permeability [50]. Kaushik et al. further validated these interfacial mechanisms at the macro-scale, demonstrating a >17% increase in recovery coupled with significant injection pressure reduction [51].

## 4. Current Challenges and Future Prospects

Despite the enormous potential and ongoing field applications of nanoemulsion oil displacement technology, further optimization faces a critical barrier: the lack of explicit structure–activity relationships. This restricts rational formulation design, making it necessary to critically assess current challenges to transition from empirical applications to scientifically guided deployments.

The current challenges are mainly reflected in three aspects: 1. Unclear structure–activity relationships restrict rational design. While field applications exist, they often rely on empirical trials rather than on quantitative guidance. The dynamic interactions of multi-components in complex porous media, especially how specific formulation structures dictate macroscopic EOR performance, urgently require more-advanced in situ characterization and validation using pilot or field-scale data. 2. Full-chain stability in practical deployment constitutes a core test. Although large-scale preparation costs are becoming manageable, practical deployment requires survival through storage and transport stability (preventing Ostwald ripening), injection stability (withstanding high shear rates at pumps), and long-term reservoir aging (maintaining chemical stability under high temperature, high salinity, and high hardness). 3. Environmental and field implementation complexities. On the implementation side, injectivity issues such as near-wellbore plugging due to nanoparticle retention and coordination with existing waterflooding processes still require precise parameter optimization. On the environmental side, the subsurface retention of synthetic nanomaterials raises ecological concerns, making the development of bio-based biodegradable emulsifiers and the systematic evaluation and quantification of nanomaterial migration risks prerequisites for sustainable development.

Looking to the future, research should systematically advance in several directions: Clarifying structure–activity relationships is the foundation, which requires coupling advanced in situ characterization with pilot/field-scale data to establish quantitative models from formulation to performance. Smart responsive nanoemulsion development will address full-chain stability paradoxes, realizing adaptive regulation triggered by reservoir conditions (temperature, pH, or salinity). Artificial intelligence-assisted design will accelerate formula optimization and predict reservoir aging. Ultimately, these efforts will transform current empirical applications into rationally designed, large-scale deployments.

## 5. Conclusions

As nanoemulsions are an advanced nanofluid oil displacement agent, the refined development of nanoemulsion characterization technologies and systematic deepening of oil displacement mechanism research has jointly driven the continuous expansion of enhanced oil recovery technology boundaries. Through the synergistic application of multi-scale characterization methods such as dynamic light scattering (DLS), small-angle X-ray scattering (SAXS), and cryo-transmission electron microscopy (Cryo-TEM), and interfacial tension measurement, researchers have been able to accurately analyze droplet size, interface structure, and stability mechanisms at the molecular-nano scale, providing a scientific basis for rational design. This “characterization-design-verification” closed-loop research model enables nanoemulsions to achieve multiple synergistic mechanisms such as ultra-low interfacial tension, effective wettability reversal, efficient emulsification and viscosity reduction, and structural disjoining pressure, thereby realizing precise stripping and efficient utilization of residual oil at the micro scale. At the application level, nanoemulsions provide a powerful technical weapon for overcoming the development challenges of difficult-to-recover reserves such as low-permeability, tight, and heavy oil. Their nano-scale size endows them with remarkable penetration ability to reach micro–nano-scale pore throats that conventional chemical agents cannot access, converting rock surfaces from oil-wet to strongly water-wet through wettability alteration, while using structural disjoining pressure to overcome capillary resistance, ultimately achieving significant recovery improvements. This technical advantage demonstrates significant potential for improving development efficiency in challenging reservoirs. However, further optimization and rational large-scale application still face multiple challenges: unclear structure–activity relationships restrict the transition from empirical formulation to rational design; full-chain stability (including storage, transport, injection stability, and long-term reservoir aging) needs reliable assurance; dynamic interactions of multi-components in complex porous media still require in situ characterization and validation with pilot/field-scale data; and potential ecological risks as well as on-site injectivity issues are yet to be systematically evaluated and optimized. These bottleneck problems restrict the rational expansion of the technology, urgently requiring breakthroughs in clarifying structure–activity relationships and engineering technology innovation for coordinated solutions.

Looking forward, the development of nanoemulsion oil displacement technology will focus on several trends: clarifying structure–activity relationships through multi-scale characterization and field data to guide rational design, developing smart responsive systems to solve full-chain stability paradoxes, and utilizing artificial intelligence to accelerate formula optimization and predict reservoir aging. Ultimately, transitioning from empirical applications to rationally designed, large-scale deployments will be the touchstone for verifying technology value. Nanoemulsion technology is expected to play an increasingly critical role in future oilfield development, promoting efficient extraction alongside environmentally friendly practices under the “green extraction” concept.

## Figures and Tables

**Table 1:** Comprehensive comparison of nanoemulsion characterization techniques.

Methods	Principle	Measured Parameters	Advantages	Limitations
Dynamic Light Scattering (DLS)	Analyzes intensity fluctuations of scattered light from Brownian motion	Hydrodynamic diameter, polydispersity index (PDI), zeta potential	Rapid, non-destructive, real-time monitoring	Assumes spherical particles, sensitive to dust, cannot distinguish polydisperse mixtures
Multi-angle DLS (MDLS) [19]	Extrapolates scattering data from multiple angles	Same as DLS, improved accuracy	Higher accuracy, extended range	More-complex instrumentation, requires longer measurement time
Small-angle X-ray Scattering (SAXS) [23]	Measures X-ray scattering at small angles	Droplet size, shape, interface layer thickness, internal structure	Statistical information on large ensemble, non-destructive, provides interface structure	Requires synchrotron source for high resolution, complex data analysis
Nanoparticle Tracking Analysis (NTA) [25]	Tracks Brownian motion of individual particles	Particle size distribution, concentration	Direct visualization of individual particles, higher precision than DLS	Limited concentration range, longer analysis time
Cryo-transmission Electron Microscopy (Cryo-TEM) [28]	Rapid freezing preserves liquid structure, electron imaging	Morphology, droplet size, spatial distribution	Avoids drying artifacts, true liquid-state imaging, atomic resolution	Expensive, complex sample preparation, limited field of view
In Situ Liquid TEM [29]	Samples in liquid cell, real-time imaging	Dynamic processes, size evolution, aggregation	Avoids drying artifacts, true liquid-state imaging, atomic resolution	Expensive, complex sample preparation, limited field of view
Atomic Force Microscopy (AFM) [31]	Scans sample surface with sharp probe	3D morphology, surface roughness, mechanical properties	Works in liquid/air, measures mechanical properties, no coating needed	Slow scanning speed, limited field of view, tip convolution effects
Interfacial Tension (IFT) Measurement [35]	Pendant drop/du Noüy ring methods	Oil–water IFT, interfacial rheology	key EOR parameter, quantifies reduction efficiency	Requires careful temperature control, sensitive to contamination
Contact Angle Measurement [22]	Optical measurement of droplet profile	Wettability, surface energy	Direct wettability assessment, simple method	Surface roughness affects accuracy, requires representative samples
Zeta Potential Analysis [44]	Electrophoretic mobility in electric field	Surface charge, electrostatic stability	Predicts colloidal stability, quick measurement	Sensitive to ionic strength, pH-dependent, dilution effects
Interfacial Shear Rheology [22]	Oscillatory deformation of interface film	Interfacial viscosity, elasticity, film strength	Evaluates interface film quality, predicts emulsion stability	Specialized equipment, sensitive to contamination, complex interpretation
Fourier-Transform Infrared Spectroscopy (FTIR) [40]	Absorption of IR light by functional groups	Chemical composition, molecular interactions	Identifies components, reveals intermolecular interactions	Water interference, limited quantitative accuracy
Nuclear Magnetic Resonance (NMR) [41]	Nuclear spin in magnetic field	Pore structure, oil saturation, molecular dynamics	Non-destructive, quantifies oil distribution, pore-scale analysis	Expensive, requires D_2_O for water signal, complex interpretation
X-Ray Diffraction (XRD) [42]	X-ray scattering by crystal lattice	Crystal structure of nanoparticles	Identifies crystalline phases, monitors structural changes	Only for crystalline materials, limited to solid components
Thermogravimetric Analysis (TGA) [43]	Mass loss with temperature increase	Thermal stability, component content	Quantitative composition, thermal behavior assessment	Destructive, cannot identify components directly

## Data Availability

No new data were created or analyzed in this study. Data sharing is not applicable to this article.

## Abbreviations

The following abbreviations are used in this manuscript:

EOR	Enhanced oil recovery
O/W	Oil-in-water
W/O	Water-in-oil
DLS	Dynamic light scattering
PDI	Polydispersity index
SPN	Surfactant–polymer–nanoparticle
PHPA	Partially hydrolyzed polyacrylamide
TEM	Transmission electron microscopy
SEM	Scanning electron microscopy
Cryo-TEM	Cryo-transmission electron microscopy
HR-TEM	High-resolution transmission electron microscopy
AFM	Atomic force microscopy
IFT	Interfacial tension
FTIR	Fourier-transform infrared spectroscopy
NMR	Nuclear magnetic resonance
XRD	X-ray diffraction
TGA	Thermogravimetric analysis
ICP-OES	Inductively coupled plasma optical emission spectroscopy
XPS	X-ray photoelectron spectroscopy
